# Impact of Islet Transplantation on Type 1 Diabetes-Related Complication: A Systematic Review

**DOI:** 10.3389/ti.2025.15091

**Published:** 2025-11-12

**Authors:** Karim Gariani, Andrea Peloso, Fadi Haidar, Rohan Kumar, Charles-Henri Wassmer, Marika Morabito, Nicerine Krause, Philippe Compagnon, Ekaterine Berishvili, Thierry Berney

**Affiliations:** 1 Division of Endocrinology, Diabetes, Nutrition and Patient Therapeutic Education, Geneva University Hospitals, Geneva, Switzerland; 2 Diabetes Center of the Faculty of Medicine, University of Geneva Medical School, Geneva, Switzerland; 3 Division of Transplantation, Department of Surgery, Geneva University Hospitals, Geneva, Switzerland; 4 Division of General Surgery, Department of Surgery, Geneva University Hospitals, Geneva, Switzerland; 5 Division of Nephrology, Department of Medicine, Geneva University Hospitals, Geneva, Switzerland; 6 Department of Surgery, University of Insubria, Varese, Italy; 7 Laboratory of Tissue Engineering and Organ Regeneration, Department of Surgery, University of Geneva, Geneva, Switzerland; 8 Cell Isolation and Transplantation Center, Department of Surgery, Geneva University Hospitals and University of Geneva, Geneva, Switzerland; 9 Institute of Medical and Public Health Research, Ilia State University, Tbilisi, Georgia; 10 Cell Isolation and Transplantation Centre, Department of Surgery, Geneva University Hospitals, Geneva, Switzerland; 11 Ilia State University School of Medicine, Tbilisi, Georgia; 12 Division of Nephrology, Immunology and Transplantation, Hôpital Edouard Herriot, Hospices Civils de Lyon, Lyon, France

**Keywords:** cardiovascular disease, islets transplantation, diabetes, neuropathy, nephropathy

## Abstract

Islet transplantation is a valuable therapy for selected type 1 diabetes mellitus (T1DM) patients, especially those with recurrent severe hypoglycemia, glycemic variability, or impaired hypoglycemia awareness. It improves glycemic control and protects against hypoglycemic episodes. Beyond glucose regulation, islet transplantation may mitigate diabetes-related microvascular and macrovascular complications. We conducted a systematic review to assess its impact on vascular outcomes in T1DM, focusing on islet transplantation alone (ITA) and islet-after-kidney transplantation (IAK). We included studies that quantitatively assessed vascular complications after ITA or IAK in adults with T1DM. Eligible studies compared pre-and post-transplant outcomes or posttransplant outcomes with control groups receiving standard treatment. Twenty-five studies (1,373 patients) evaluated microvascular and macrovascular outcomes using eGFR, ophthalmic e xams, and nerve conduction studies. Islet transplantation was associated with stabilization or improvement in most microvascular complications and longterm renal function preservation. While macrovascular data were less frequent, improvements in vascular health markers such as reduced procoagulant states and atherosclerosis progression were reported, suggesting possible reductions in cardiovascular events and mortality, though data remain limited. Islet transplantation shows clear benefits for microvascular complications and potential advantages for macrovascular outcomes, alongside its established role in improving glycemic stability and quality of life.

**Systematic Review Registration**: PROSPERO Identifier CRD420251036400.

## Graphical Abstract

**Figure:**
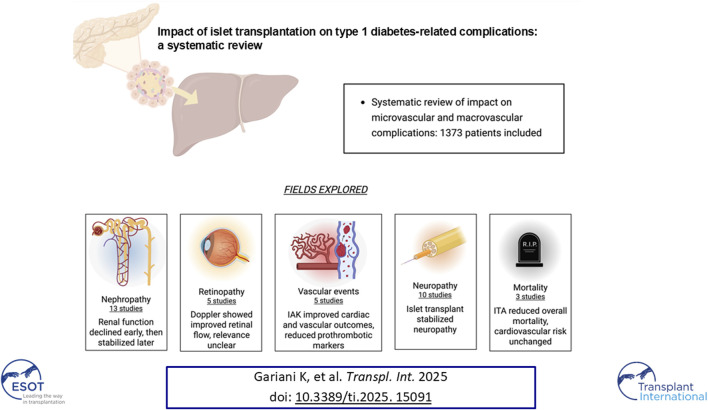

## Introduction

The landmark Diabetes Control and Complications Trial (DCCT) established that intensive glucose management in individuals with type 1 diabetes mellitus (T1DM) significantly reduces the incidence of microvascular complications. The subsequent Epidemiology of Diabetes Interventions and Complications (EDIC) study, which extended the follow-up of DCCT participants, further demonstrated that sustained glycaemic control confers long-term protection not only against microvascular complications but also against macrovascular complication events, including myocardial infarction, stroke, and cardiovascular (CV) mortality [1].

Islet transplantation (ITx) has emerged as a valuable treatment option for patients with unstable T1DM. Over the past three decades, substantial progresses have been made in elucidating the mechanisms underlying the loss of functional islet mass, as well as in developing strategies to preserve and enhance islet survival [2–5]. ITx provides significant benefits, particularly for patients with problematic glycaemic profile instability [6, 7]. Recent long-term outcome data have yielded encouraging results. At 1-year post-transplantation, approximately 60% of recipients achieved insulin independence. This proportion declined to around 30% at 5 years and to approximately 20% by 10 years [8]. Nevertheless, even partial islet function may have beneficial effects on diabetes-related complications compared to complete loss of graft function.

In the Edmonton single-centre cohort of 255 patients followed for up to 20 years after ITx [8], 70% achieved sustained graft survival, with a median graft survival of 5.9 years and insulin independence rates of 61% at 1 year and 8% at 20 years. Prolonged graft survival was significantly associated with older recipient age, longer diabetes duration, lower baseline insulin requirements, the combined use of anakinra and etanercept (adjusted odds ratio 7.5), and a BETA-2 score of 15 or higher at 6–12 months after transplantation (adjusted odds ratio 4.1).

Nevertheless, a considerable number of patients who resumed insulin therapy retained partial graft function, resulting in improved glycaemic control and a marked reduction in the frequency and severity of hypoglycaemic episodes. These findings underscore the potential of ITx to provide durable metabolic benefits and to the long-term potential of ITx to enhance metabolic stability and quality of life for patients with T1DM.

T1DM is associated with a range of long-term complications, including microvascular diseases such as diabetic retinopathy, neuropathy, and nephropathy, as well as cardiovascular diseases (CVD). After approximately a decade of disease progression, microvascular complications are observed in around 50% of individuals, while approximately 6% experience macrovascular involvement [9]. Although hyperglycaemia may be managed through exogenous insulin therapy, this does not fully replicate the endogenous, finely tuned regulation of blood glucose. ITx provides an established therapeutic approach aimed at restoring insulin secretion, thereby facilitating improved glycaemic control. This approach holds the potential not only to mitigate, but in some cases to prevent, the progression of diabetes-related complications.

Considering this, we undertook a systematic review of the literature to assess the impact of ITx on both micro- and macrovascular complications associated with T1DM.

## Methods

### Data Sources and Searches

The approach for search strategy, study selection, and data extraction and analysis was guided by a pre-defined protocol registered with the International Prospective Register of Systematic Reviews (PROSPERO, CRD420251036400). This systematic review adhered to the Meta-analysis of Observational Studies in Epidemiology (MOOSE) guidelines [10].

We systematically identified all studies providing a quantitative assessment of diabetes-related complications after islet transplant alone (ITA) or islet-after-kidney (IAK). The literature search encompasses Medline records from 1966 to April 2025. A comprehensive search strategy was conducted using the following combination of keywords and MeSH terms: (islet OR IAK OR ITA) AND (graft OR allograft OR transplant*) AND (microvascular OR macrovascular OR microvascular complication* OR macrovascular complication* OR chronic complication* diabet* complication* OR diabetic nephropathy OR nephropathy OR albuminuria OR microalbuminuria OR macroalbuminuria OR diabetic kidney disease OR kidney disease OR renal disease OR maculopathy OR retinopathy OR diabetic retinopathy OR neuropathy OR diabetic peripheral neuropathy OR diabetic neuropathy OR peripheral neuropathy OR sensory neuropathy OR peripheral arterial disease OR Peripheral Vascular Diseases OR diabetic foot OR foot ulcer OR amputation OR atherothrombo* OR stroke OR CVD OR myocardial infarction OR ischaemic heart disease).

No restriction was placed on publication date or language during the literature search. In addition, the reference lists of all retrieved articles were manually screened to identify further relevant studies. Two investigators (KG and AP) independently assessed titles, abstracts, and full-text articles. Discrepancy regarding study inclusion were resolved through discussion between the two reviewers, with the involvement of a third author (TB) when consensus could not be reached.

### Eligibility Criteria

We included observational, prospective studies that investigated the progression of diabetes-related complications after transplantation in adults with T1DM who had undergone IAK or ITA. Diabetes-related complications were defined as the occurrence of nephropathy, retinopathy, neuropathy, peripheral arterial disease, lower-limb amputations, foot ulcers, stroke and myocardial infarction. Studies were excluded if they were case reports, letters to the editor, or investigations conducted on animal models.

### Data Extraction

Data extraction was performed by KG in accordance with predefined criteria and independently verified for accuracy by AP. Extracted data included demographic and clinical characteristics of the study population (such as mean age, sex, study design, and time elapsed since transplantation), study characteristics (including country of origin, study design, sample size, and duration of follow-up. Outcomes of interest were defined as diabetes-related complications.

### Data Synthesis and Methodological Quality Rating

Owing to substantial heterogeneity in the methodologies employed to assess diabetes-related complications—ranging from generic to disease-specific measures—as well as marked variability in study designs and the limited number of studies evaluating certain complications, neither meta-analysis nor direct comparison of outcomes was undertaken.

The methodological quality of the included studies was appraised using the Newcastle–Ottawa Scale (NOS) [11]. This scale evaluates three key domains: selection of study participants (up to four points), comparability of study groups (up to two points), and ascertainment and reporting of outcomes (up to three points), with a maximum total score of nine. Based on total score, studies were classified as poor (0–3), fair (4–6), or good (7–9). Studies scoring ≤3, indicating significant methodological limitations were excluded from further analysis. Scoring was performed by KG and independently verified for accuracy by AP. The findings were therefore synthesised narratively, without the generation of pooled estimates for diabetes-related complications.

## Results

### Characteristics of Included Studies, Methodological Quality

**FIGURE 1:**
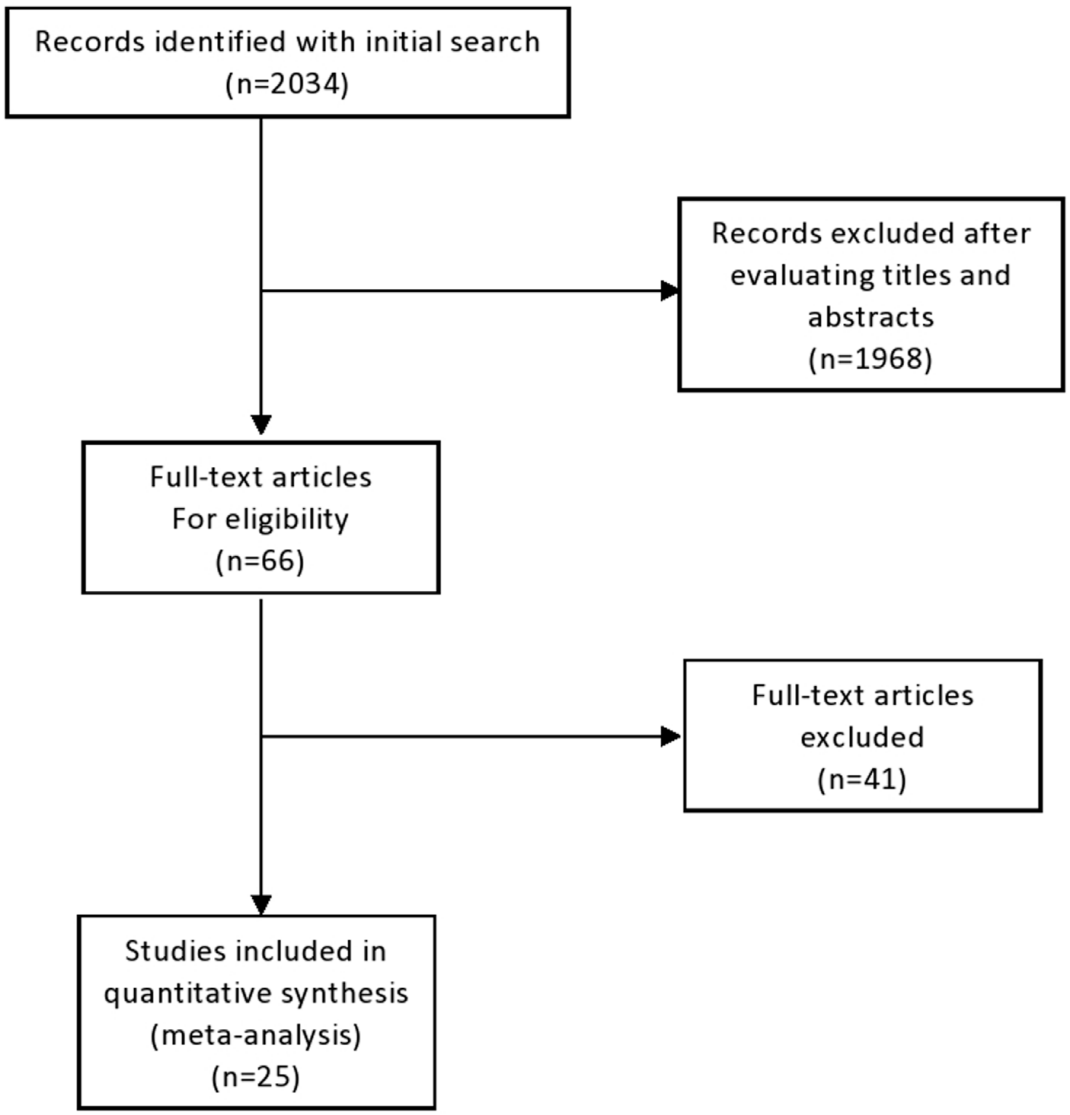
Flow-chart of study selection.

Our search strategy yielded 2,034 unique records, of which 1,968 following title and abstracts screening. A total of 66 full-text articles were assessed for eligibility. Upon detailed evaluation, 30 studies met the predefined inclusion criteria. Five articles were excluded due to overlapping patient populations and smaller sample sizes relative to included studies. Finally, 25 studies, encompassing a cumulative total of 1,373 patients, were included in this systematic review. [12–34]. The study selection process is summarized in Figure 1.

Among the included studies, 13 were conducted in Europe, 10 in North America and 3 in Oceania. In terms of study design, 14 were prospective cohort studies, 8 were retrospective analyses, and 2 were cross-sectional studies. Detailed characteristics of the included studies, along with the diagnostic methodologies employed, are described in Table 1.

**TABLE 1:** Main characteristics of the included studies.

Author, year	Design	Country	Type of transplantation	Time assessmeent after tx	Transplanted individuals (N)	Age (years)	Female (%)	Diabetes duration before (tx)	Complication assessed	Comparison group	NOS score
Alhaidar [12]	P	USA	ITA	75 months	13	44.5	69	NA	Diabetic neuropathy	Before ITA	6
Andres [13]	R	Switzerland	ITA and IAK	12 months	5 ITA and 5 IAK	43	50	NA	Diabetic nephropathy	Combined groups (ITA and IAK) before transplantation	6
Barton [14]	P	USA, Canada, France, Switzerland, Italy, Australia	ITA (85%) and IAK/SIK (15%)	5 years	677	44.3	59.2	28.8	Diabetic nephropathy	Combined groups (ITA, IAK, SIK) before transplantation	8
D’Addio [15]	P	Italy	ITA	15 months	12	36.9	67	23.3	Prothrombotic factors, platelet function and ultrastructure	Type 1 diabetic patients and healthy control individuals	7
Danielson [16]	P	USA	IAK	12 months	15	49	87	30.1	Carotid intima-media thickness	Before IAK	7
Del Carro [17]	P	Italy	IAK	53 months	18	41.8	56	26.3	Diabetic neuropathy	Kidney transplantation alone and before IAK	7
Deshmukh [18]	R	Australia	ITA	4 years	8	NA	NA	NA	Cardiac autonomic neuropathy (CAN)	Type 1 diabetes individuals	7
Fensom [19]	Crossectional	Canada	ITA	74 months	30	44		29	Diabetic neuropathy	Medical therapy	8
Fiorina [20]	P	Italy	ITA	7 years	24	41.9	NA	27.2	Diabetic nephropathy	T1D individuals with unsuccessful islet transplantation	7
Fiorina [21]	P	Italy	IAK	7 years	37	41.8	NA	27.1	Cardiovascular death, endothelial injury and atherothrombotic profile	T1D patients still on hemodialysis	7
Fiorina [22]	P	Italy	IAK	3 years	17 IAK 25 KTA	48.6	45.2	31	Carotid intima-media thickness Left ventricular function	Before IAK	7
Hering [35]	P	Canada and USA	ITA	24 months	48	48.4	60	28.5	Diabetic nephropathy	Before ITA	8
Lee [23]	P	USA	ITA	12 months	8	NA	NA	NA	Diabetic neuropathy and diabetic retinopathy	Before ITA	6
Lehmann [24]	P	Switzerland	SIK/IAK	13 years	38	51.8	50	37	Diabetic nephropathy	Combined SPK/PAK after transplantation	7
Maanaoui [25]	R	France	IAK	10 years	40	46.1	43	NA	Diabetic nephropathy	Kidney transplantation alone	8
Nijhoff [34]	R	Netherlands	IAK	24 months	13	50.9	38	35,5	Diabetic nephropathy	Before ITx	7
O’Connell [26]	P	Australia	ITA	12 months	17	49.8	82		Diabetic nephropathy	Before ITA	6
Palmer [36]	R	Australia	ITA	4.7 years	16	NA	NA	NA	Cardiovascular autonomic neuropathy	Before ITA	6
Perrier [27]	R	France	ITA, IAK	11.7 years	61 ITA and 45 IAK	48	44.3	32	First occurrence of a composite criterion composed of mortality, transient ischemic stroke, nonfatal stroke, nonfatal myocardial infarction, amputation or dialysis.	T1D control patients for ITA and T1D + KTA for IAK.	8
Rickels [28]	R	USA, Canada	ITA and IAK	4.55 years of follow-up in the ITA group and 3.43 years of follow-up in the ITA group	48 ITA and 24 IAK	48.6	55.6	33	Diabetic nephropathy	Before ITA for individuals with ITA and before IAK for individuals with IAK	8
Ryan [29]	R	Canada	ITA	5 years	65	42.9	57	27.1	Diabetic nephropathy, diabetic neuropathy and diabetic retinopathy	Before ITA	8
Tekin [30]	R	USA	ITA	9.2 years	4	44.3	75	30	Diabetic nephropathy, diabetic neuropathy, and diabetic retinopathy	Before ITA	6
Thompson [31]	Crossectinal	Canada	ITA	66 months	29	NA	NA	NA	Diabetic nephropathy, diabetic neuropathy and diabetic retinopathy	DT1 individuals intensively medically treated	7
Vantyghem [32]	P	France	ITA, IAK	5 years	21	NA	NA	NA	Diabetic neuropathy and CAN	Combined groups (ITA,IAK) before transplantation	6
Venturini [33]	P	Italy	ITA	12 months	10	38	NA	24.9	Diabetic retinopathy	Before ITA and DT1 patients on waiting list for transplantation	7

C, cross-sectional study; CAN, cardiac autonomic neuropathy; IAK, islet after kidney; ITA, islet transplant alone; NA, non-available; P, prospective study; PAK, pancreas after kidney transplant; R, retrospective study; SIK, simultaneous islet-kidney transplantation; SPK, simultaneous pancreas-kidney transplant.

### Study Quality

According to the NOS scale performed for a critical appraisal, the overall methodological quality of the studies was deemed to be satisfactory, with an average NOS score of 7.0 out of a maximum of 9, indicating a generally robust standard of reporting and design across the included literature. Specifically, 7 study achieved a score of 8, reflecting a high level of methodological rigour, 11 studies received a score of 7, denoting good quality with minor limitations, while 7 studies scored 6, suggesting moderate quality with some potential risk of bias in one or more domains. Importantly, no study was excluded on the basis of methodological weakness, as none score below the exclusion threshold of 2 points. This overall quality distribution supports the reliability of the findings synthesised in the review and lends confidence to the narrative interpretation of the reported outcomes (Table 1).

### Impact on Microvascular Complications

#### Diabetic Nephropathy

**FIGURE 2:**
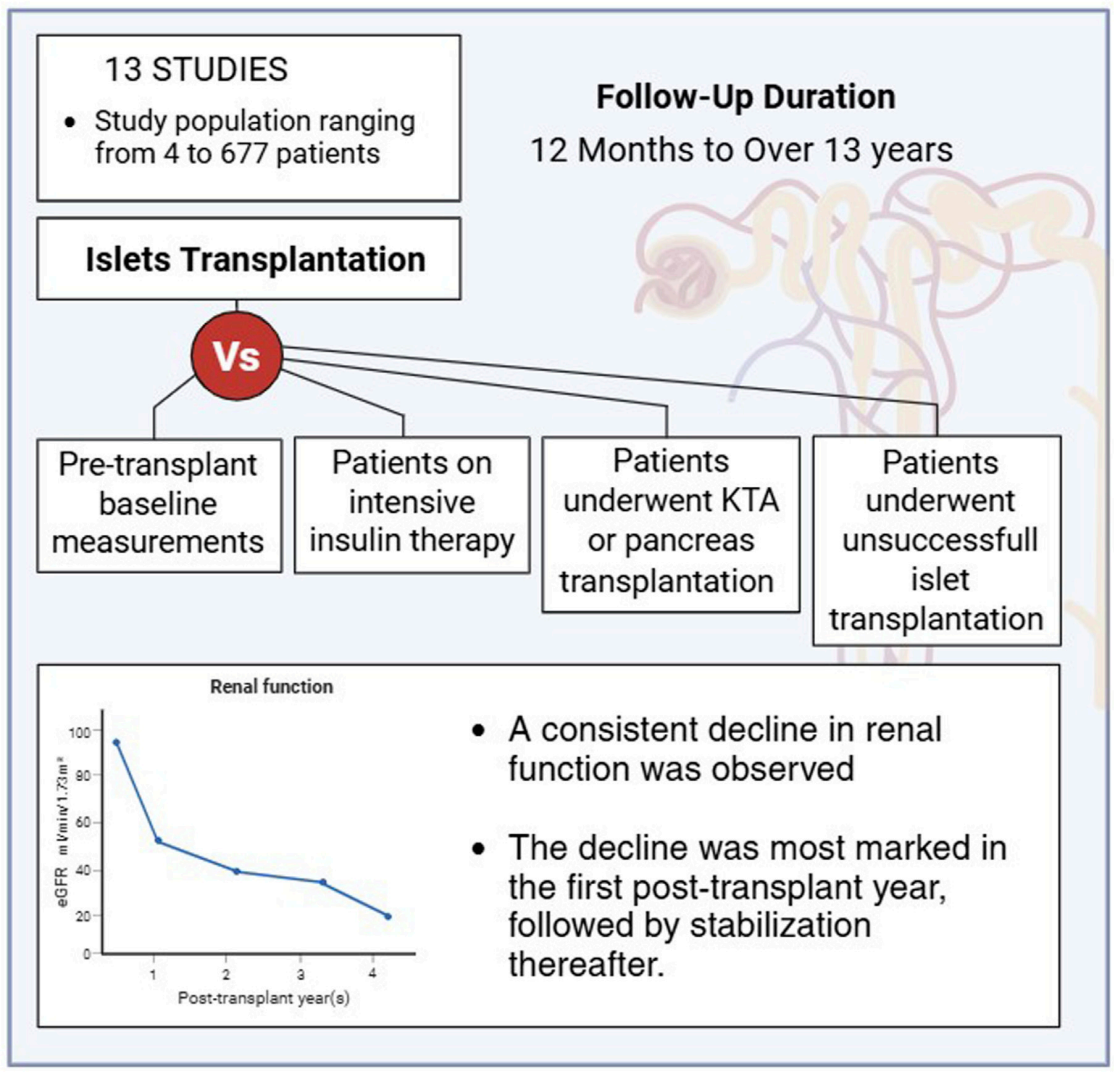
Summary of 13 studies with follow-up durations ranging from 12 months to 13 years, showing an initial decline in renal function during the first year post-transplantation, followed by stabilization in subsequent years.

13 studies evaluating the progression of renal function and diabetic nephropathy following ITx were identified. The sample size across studies varied substantially, ranging from 4 to 677 participants, as did the duration of follow-up, which spanned from 1 to 13 years (Table 1). Renal function at the end of the follow-up assessed either in comparison to the pre-transplant baseline measurements or to various control groups, including individuals managed with intensive insulin therapy (basal-bolus regimens), recipients of kidney transplantation alone (KTA), recipients of pancreas transplantation, or individuals in whom ITx was unsuccessful. Among studies comparing renal function before and after transplantation, with follow-up periods ranging from 1 to over 9 years, a decline in renal function was consistently observed (Figure 2). This decline appeared to be more pronounced during the first post-transplant year, with reductions in eGFR reaching up to 10 mL/min, followed by stabilization in the subsequent years [26, 28, 35].

To reduce the potential confounding impact of immunosuppressive therapy on renal outcomes, one study compared 40 T1D transplant recipients who received IAK with 80 individuals who underwent kidney transplant alone (KTA). The IAK group demonstrated a significantly slower rate of renal function decline relative to the KTA group [25].

Furthermore, a separate study comparing 45 IAK and 61 ITA recipients reported a significant lower incidence of dialysis initiation among the IAK cohort, thereby reinforcing the evidence of the renal protective effect of IAK over ITA [27].

Patients with unsuccessful ITx exhibited a higher incidence of renal graft loss and a more pronounced increase in the microalbuminuria index compared to individuals in the IAK group who maintained functioning grafts [20]. Finally, when compared with a cohort of 8 T1DM patients treated with intensive insulin therapy, 29 recipients of IT showed an attenuated decline in eGFR [31].

#### Diabetic Retinopathy

**FIGURE 3:**
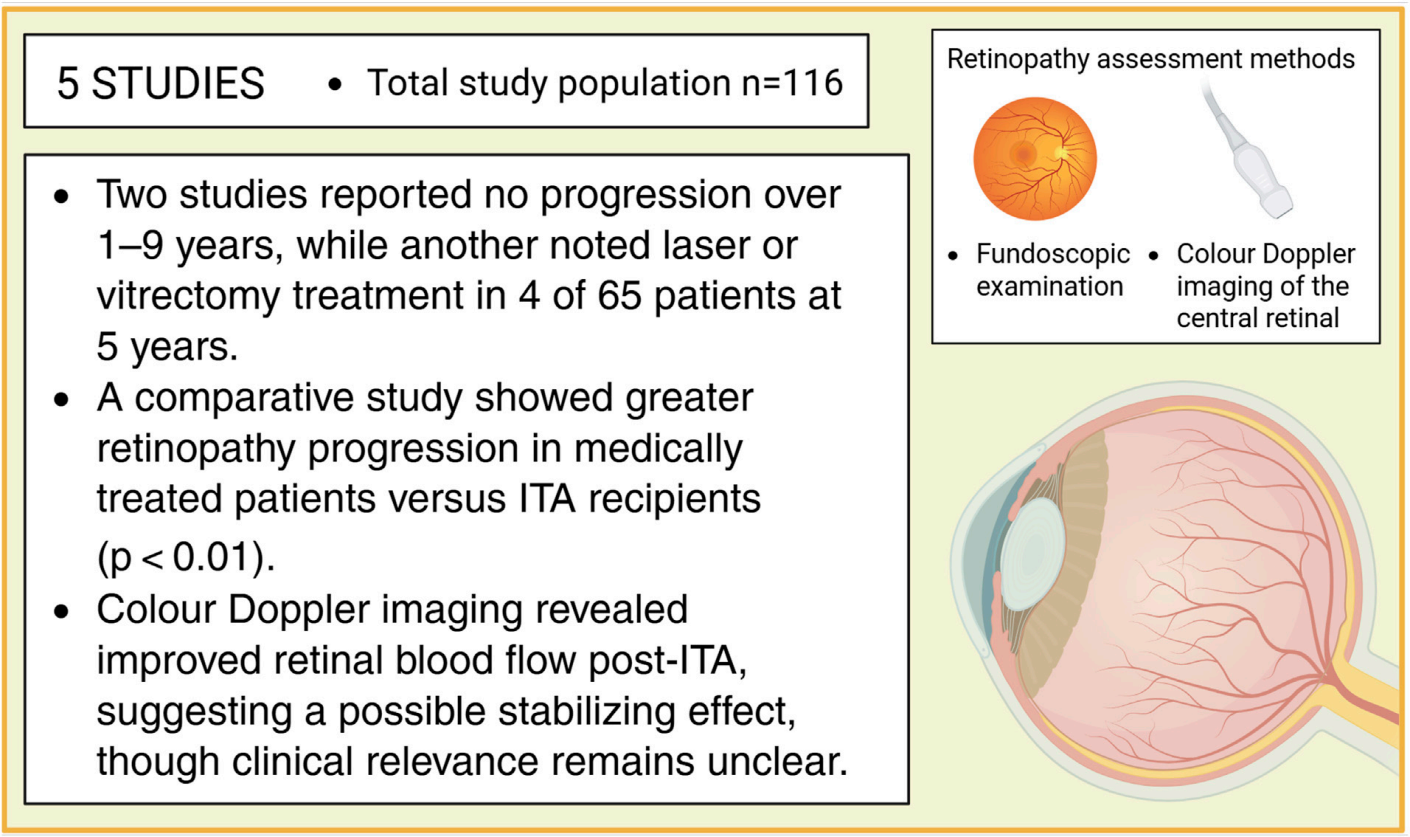
Impact of islet transplantation on the progression of diabetic retinopathy, showing a tendency toward stabilization and more favorable outcomes compared to patients treated with medical therapy alone.

Data on diabetic retinopathy progression was reported in 5 studies, including a total of 116 patients (Figure 3). All studies exclusively included patient who had undergone ITA, with baseline prevalence of diabetic retinopathy ranging from 50% to 80%. Retinopathy assessment methods included fundoscopic examination or colour Doppler imaging of the central retinal arteries and veins. In longitudinal analyses comparing retinopathy status before and after ITA, 2 studies reported no progression of diabetic retinopathy, indicating clinical stability over follow-up periods ranging from 1 to 9 years [23, 30]. Another study, with a 5-year follow-up, showed that, among 65 patients, 4 required treatments with laser photocoagulation or vitrectomy [29]. A comparative study involving a cohort of 29 individuals evaluated retinopathy progression in ITA recipients versus those receiving medical treatment alone and demonstrated a significantly greater progression of retinopathy in the medically treated group (p < 0.01) [31]. In a separate study using colour Doppler imaging to assess retinal vasculature, a significant improvement in blood flow velocities both in the central retinal artery and vein was observed at 12 months post-ITA in 10 recipients. This finding suggests a potential stabilising or protective effect of ITA on the progression of diabetic retinopathy. In contrast, among patients with T1DM followed for 1 year, no change was observed in the central retinal artery, while a trend towards reduced flow velocity was noted in the central retinal vein, possibly indicative of disease progression [33]. The precise clinical significance of these haemodynamic changes, however, remains to be determined.

#### Diabetic Neuropathy

**FIGURE 4:**
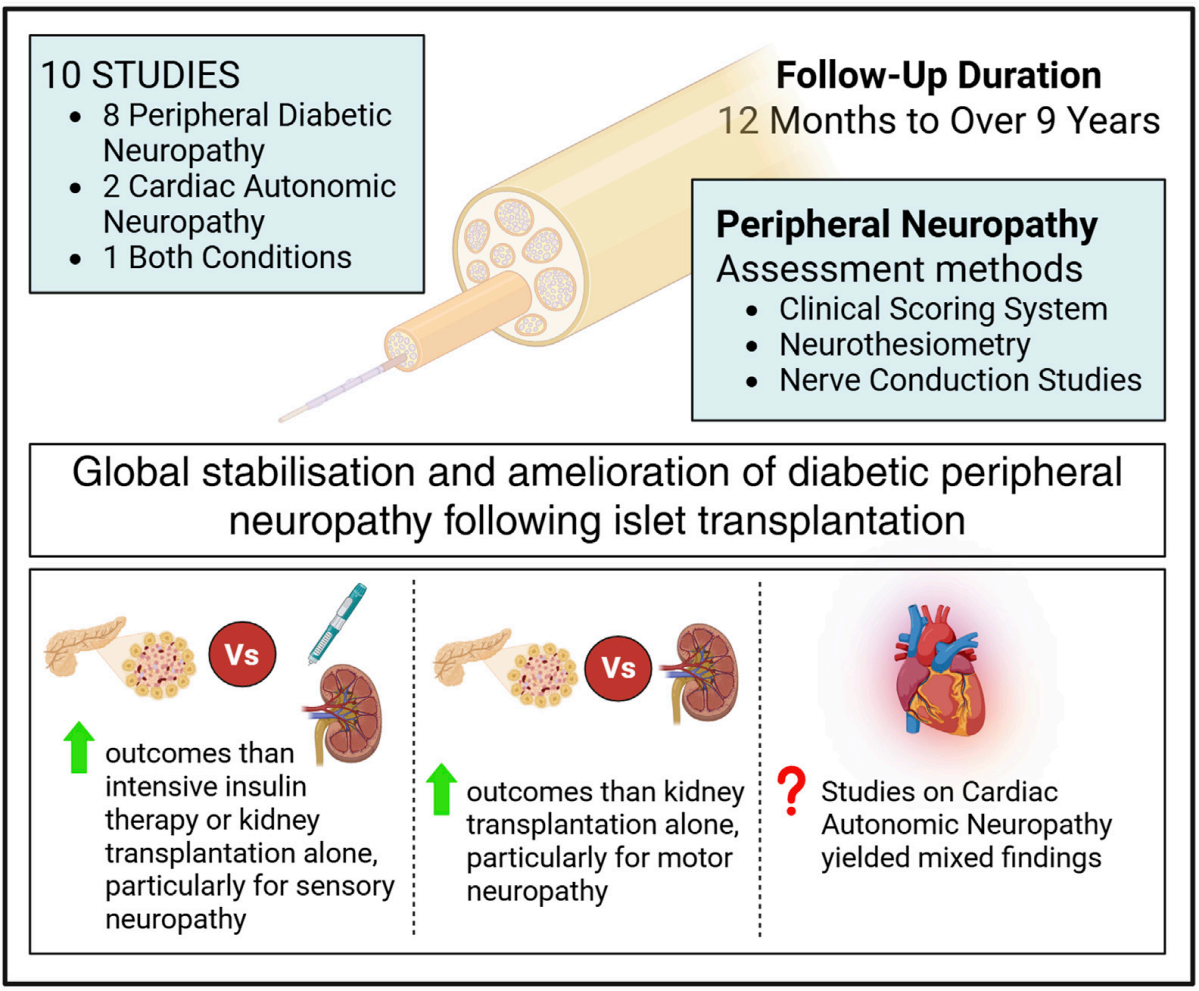
Effect of islet transplantation on diabetic neuropathy, based on 10 studies with follow-up durations ranging from 12 months to 9 years, showing a general trend toward stabilization.

A total of 10 studies assessing the progression of diabetic neuropathy after ITx were identified. Of these, 8 focused specifically on peripheral diabetic neuropathy, 2 addressed cardiac autonomic neuropathy (CAN), and 1 investigated both conditions. Follow-up duration ranged from 12 months to over 9 years. Assessment of diabetic peripheral polyneuropathy employed a variety of methodologies including clinical scoring systems, neurothesiometry, and nerve conduction studies (NCS) (Figure 4). Across these studies, a general trend towards stabilization of diabetic peripheral neuropathy after transplantation was observed, with some patients showing improvements in conduction parameters.

Notably, two studies comparing the progression of neuropathy in T1DM patients undergoing ITx versus those receiving intensive insulin therapy (IIT) reported differing outcomes: while neuropathy tended to improve in the IIT group, a worsening was observed in the non-transplanted T1DM controls [19, 31].

In the study comparing the progression of diabetic neuropathy between 18 ITx patients and 9 undergoing KTA, the aim was to mitigate the potential confounding influence of immunosuppressive therapy, known for its possible neurotoxic effects. Diabetic neuropathy was present in all participants at baseline, prior to transplantation. Neuropathy progression was assessed using NCS scores, sensory action potentials (SAP), and compound motor action potential (CMAP) amplitudes. The ITx group showed an improvement in the NCS score over time, whereas no significant change was observed in the KTA group after 53 months of follow-up.

Furthermore, improvements in SAP and CMAP amplitudes were observed in the ITx group, while both parameters declined in the group undergoing KTA [17].

Regarding the type of neuropathic involvement, sensory neuropathy showed a tendency to improve relative to the pre-transplant baseline values, while motor function remained stable [12, 32]. Moreover, when compared with control groups consisting of patients receiving either medical treatment or KTA, patients undergoing ITx demonstrated significantly more favorable outcomes [17, 19].

CAN was assessed in three studies. In a study evaluating heart rate variability via a 24-h Holter monitor, no significant difference was observed between a group of patients 4 years after ITA and a control group of T1DM patients [18].

Two additional studies examined CAN in patients both before and after ITx. In a cohort of 16 subjects, a significant reduction in resting heart rate was observed nearly 5 years following ITA, compared to baseline values prior to transplantation, thus suggesting an improvement in CAN [36]. In contrast, another study involving 21 subjects, no significant difference in CAN was observed 5 years post-transplantation [32].

#### Macrovascular and Coagulation Parameters

**FIGURE 5:**
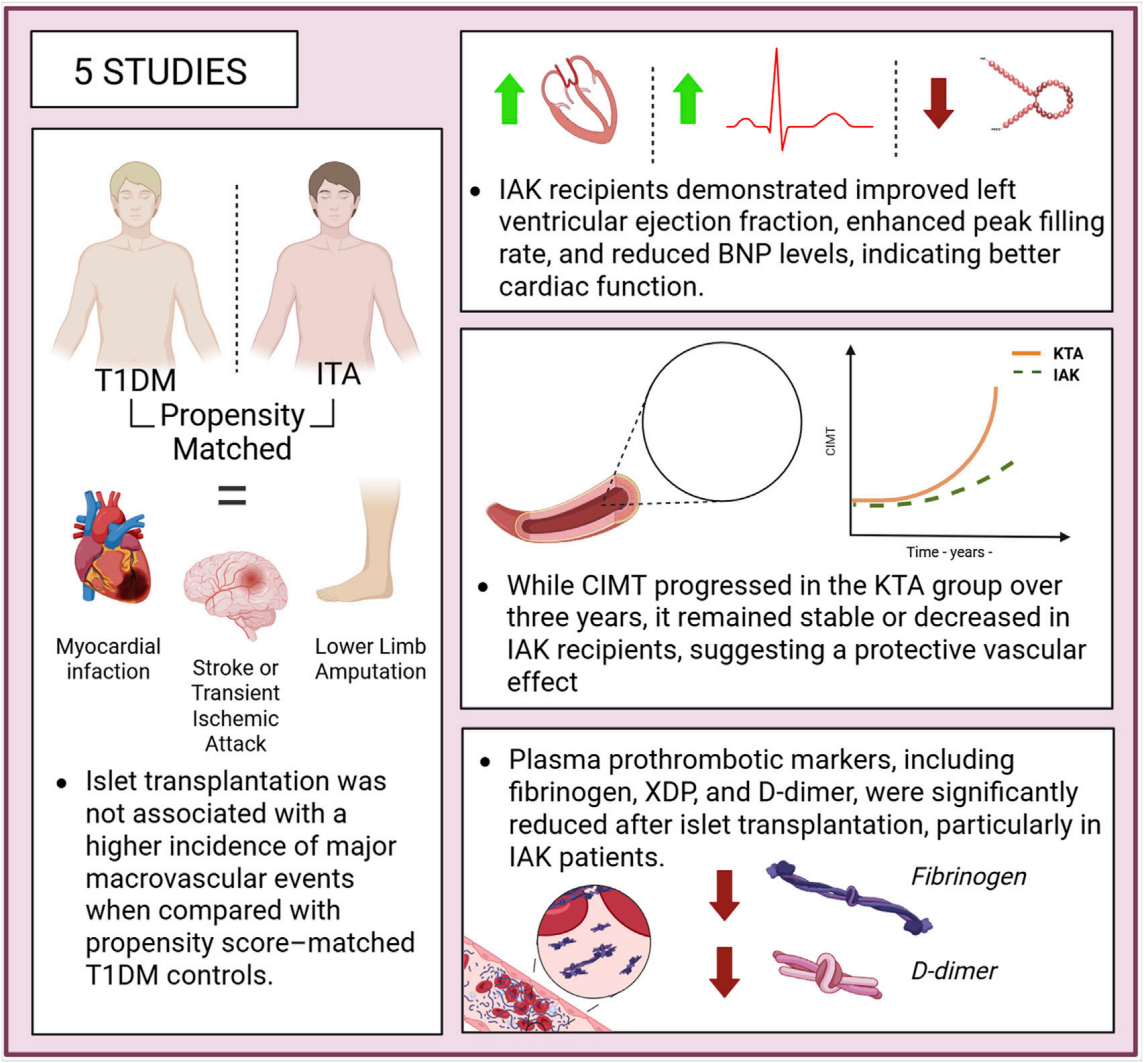
Cardiovascular outcomes following islet transplantation, showing improved myocardial function, stabilization or regression of carotid intima-media thickness (CIMT), reduced pro-thrombotic markers, and no increase in macrovascular events compared to matched patients with type 1 diabetes managed with medical therapy.

Five studies have explored the impact of ITx on outcomes related to mortality, CVD, atherosclerosis development, left ventricular function, prothrombotic status, and endothelial injury. A multicentre study comparing 61 ITA recipients with propensity score-matched 610 T1DM patients reported no significant differences in the incidence of major macrovascular events, such as myocardial infarction, stroke, transient ischemic attack, or lower-limb amputation. Interestingly, data from the same study suggested a higher risk of myocardial infarction in the 45 IAK individuals (Figure 5) [27].

In another study involving 15 patients, a reduction in carotid intima-media thickness (CIMT) was observed 12 months post-transplantation, as assessed by arterial ultrasound, compared to pre-transplant measurements. Among the seven patients re-evaluated at 50 months, both common and internal CIMT remained significantly lower than at baseline [16]. A separate study compared changes in CIMT and left ventricular function between patients undergoing 17 IAK and 25 KTA recipients, from baseline to 3 years post-transplant. Results indicated that CIMT remained stable over the 3-year period in the IAK group, whereas the KTA group experienced a significant progression in CIMT. From a cardiological perspective, patients in the IAK group showed significant improvements in left ventricular function, as evidenced by an increased left ventricular ejection fraction, an enhanced mean peak filling rate, and a reduction in B-type natriuretic peptide (BNP) levels. In contrast, no significant changes were observed in the KTA group, indicating that both systolic and diastolic ventricular function improved in the IAK cohort, while remaining stable in the KTA group [22].

Two studies assessed the evolution of plasma prothrombotic markers following ITx. Compared to 196 T1DM subjects on haemodialysis, 37 patients with IAK showed a reduction in various atherothrombotic plasma markers, such as XDP levels or F1 and F2 fragments. Additionally, an increase in antigenic activity of protein C and ATIII levels was observed, indicating an improvement in natural coagulation activity. A significant reduction in CV mortality was also observed in the group of patients with IAK [37].

Another study, also focusing on atherothrombosis parameters, highlighted that 12 patients undergoing ITA showed near-complete normalization of platelet morphology and function. Moreover, levels of prothrombotic markers such as fibrinogen and D-dimer approached normal values, in contrast to T1DM subjects who had not undergone transplantation [15].

#### Mortality

To date, data concerning mortality following ITA or IAK remain very limited, with only three studies available. In one of these investigations, 61 ITA recipients were matched with 610 T1DM control patients and followed for over a decade. A significant reduction in all-cause mortality was observed among ITA recipients compared to their T1DM counterparts [27]. This observation should be interpreted cautiously because of the small number of events; nonetheless, it might reflect a decrease in CV incidents, as suggested by the trend of fewer myocardial infarction and strokes in the ITA group.

**FIGURE 6:**
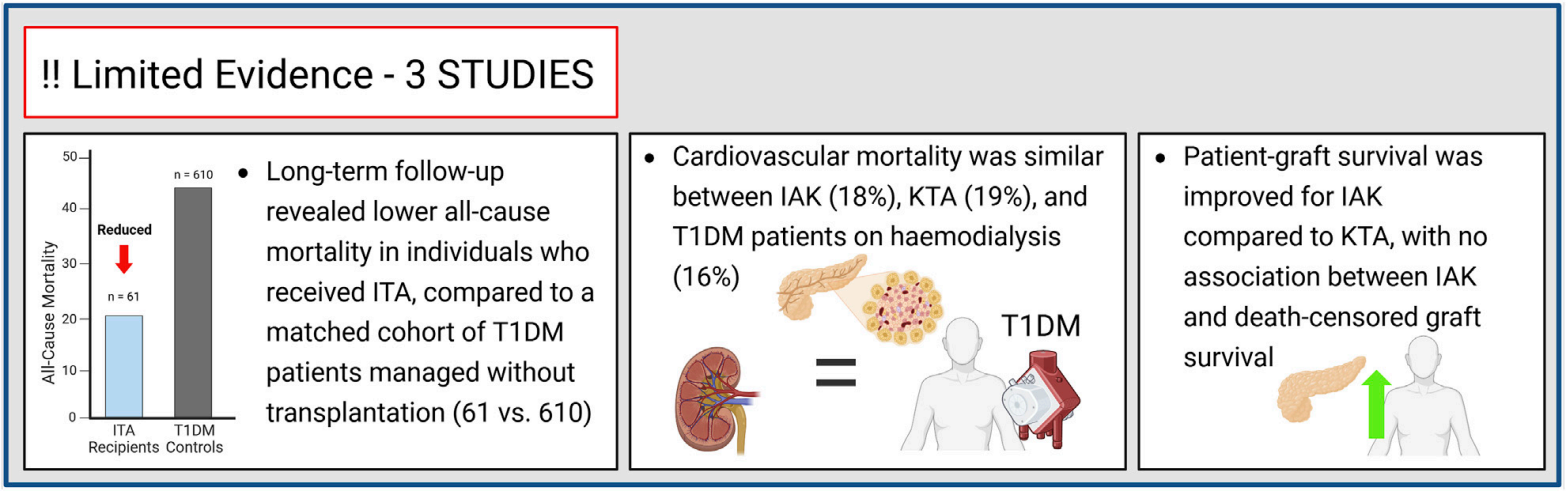
Reduced all-cause mortality following islet transplantation, compared to a matched cohort of patients with type 1 diabetes, with similar mortality rates observed between patients undergoing IAK or KTA and those receiving hemodialysis.

In the study by Maanaoui et al. [25], 40 kidney transplant recipients with type 1 diabetes who underwent IAK transplantation demonstrated significantly improved patient–graft survival compared with those who received KTA. Notably, this benefit was primarily driven by a reduction in mortality risk, rather than by an improvement in death-censored graft survival or a slower decline in renal function. Specifically, no significant association was found between IAK and death-censored graft survival (HR 0.73, 95% CI 0.30–1.89; p = 0.36), indicating that the protective effect was attributable to a reduced risk of death rather than improved renal outcomes *per se*. Importantly, CV causes and sudden death at home were the main causes of mortality (Figure 6).

The third study evaluating mortality after ITx, reported that the rate of CV death among the 37 individuals of the IAK group (18%) was comparable to that observed among the 42 patients from the KTA group (19%) and in the 196 people with T1DM undergoing haemodialysis (16%).

A lower CV mortality rate was found in the group underwent simultaneous islet and kidney transplantation (5%), comparable to the rate observed in the uremic T1DM patients who received SPK transplantation (8%). Moreover, within the ITA group, CV outcomes varied according to the success of ITx, underscoring the potential additional benefit of achieving successful and sustained islet function [37]. Of note, mortality was not considered a primary outcome in the included studies, and any interpretation should therefore be regarded as exploratory analysis.

## Conclusion

This systematic review highlights a consistent improvement in microvascular complications (and possibly in macrovascular outcomes) following ITx, whether ITA or IAK. These benefits were observed in comparison with the pre-transplant state, standard medical therapy in T1DM, and KTA. IT was found to promote stabilization and, in some cases, potential improvement, of several diabetes-related complications relative to baseline. Such improvements are particularly important for T1DM patients who suffer from several complications that impact significantly the quality of life, induce morbidity and potentially impact their life-expectancy.

These findings may, in part, be attributed to the improvement in post-transplant glycaemic control, a relationship previously observed in the DCCT. For instance, regarding diabetic neuropathy, it was present at baseline in 5.6% of patients in the conventional group and 6.8% in the intensive group. After 6.5 years of follow-up, the prevalence increased to 17.5% in the conventional group, compared to only 9.3% in the intensive group [1].

A decline in renal function is frequently observed during the initial years following transplantation, likely because of immunosuppression nephrotoxicity, followed by a stabilization of renal function [20, 26]. In particular, IAK leads to a significant improvement in clinical outcomes in the context of chronic kidney disease (CKD), including a reduction in the need for dialysis and lower mortality rates. These outcomes underscore the value of islet transplantation, beyond the observed biological impact on renal function [25]. Severe biological mechanisms may contribute to the observed improvement, or reduction, in renal function decline after transplantation. These include sustained intensive glycaemic management in some patients, improved overall glycaemic control, and the potential nephroprotective effect of C-peptide [31, 38]. Current evidence indicates that C-peptide interacts with a G-protein-coupled receptor sensitive to pertussis toxin, triggering several intracellular signalling cascades that promote the expression of genes involved in kidney protection. Additionally, studies conducted in both animals and humans have demonstrated the beneficial effects of C-peptide administration [39]. In animal models, treatment with C-peptide has been shown to significantly reduce glomerular hyperfiltration, glomerular enlargement, expansion of the mesangial matrix, and proteinuria [40]. In human subjects, C-peptide infusions have been associated with decreased hyperfiltration and lower levels of microalbuminuria. These results suggest that C-peptide exerts a protective effect on the kidneys, potentially offering benefits from islet function restoration beyond merely improving blood glucose control [41]. Nevertheless, accurately delineating the impact of ITx on renal function remains inherently challenging. Heterogeneity in baseline renal function, variations in study design, and the potential nephrotoxicity effects of immunosuppressive regimens, may confound the interpretation of post-transplant renal outcomes across available studies.

ITx has been shown to promote the stabilization of diabetic neuropathy over the medium and long term, with follow-up extending beyond 10 years. Improvements have been observed particularly in sensory neuropathy, although similar benefits have not been reported for motor neuropathy. Since hyperglycaemia is a key driver in the development and progression of diabetic neuropathy, the achievement of euglycemia, often obtained following islet transplantation, likely plays a central role in mediating the observed neurological benefits [42].

At the mechanistic level, a reduction in the expression of receptor for advanced glycation end products (RAGE) within the *vasa nervorum* was documented 4 years post-transplantation [17, 43]. This finding is of relevance, as RAGE is implicated in the pathophysiology of diabetic neuropathy. Additionally, the elevated levels of C-peptide observed after transplantation have been linked with potential improvement in nerve function [44]. C-peptide has bioactive properties, as demonstrated by preclinical studies, which have shown that its administration in animal models of diabetic neuropathy can lead to structural and functional improvements in peripheral nerves [45, 46]. Finally, the positive impact of ITx on renal function may confer an indirect benefit on neuropathy, by reducing the neuropathic damage associated with chronic kidney disease [47].

The evidence gathered in this systematic review suggests that, akin to its effects on diabetic neuropathy, ITx contributes to stabilize diabetic retinopathy. This is likely related to the improvement in glycaemic control, which may reverse endothelial dysfunction and thus enhance retinal microvascular perfusion [48]. Clinically, it has been observed that a 10% reduction in HbA1c value results in an approximate 40% reduction in the risk of diabetic retinopathy progression [49]. The post-transplant rise in circulating C-peptide levels may exert a direct beneficial effect on the retina. Experimental evidence has shown that C-peptide administration can reduce fluorescein leakage across the blood–retinal barrier, supporting a potential role in limiting vascular permeability [41]. In a small number of cases, a transient worsening of diabetic retinopathy following ITx has been observed [29, 31]. This phenomenon is likely attributable to the rapid normalisation of glycaemia, rather than being a direct consequence of the transplant itself. Such early deterioration, typically occurring within the first post-transplant year, has also been reported with other treatments that intensify glycaemic control in patients with existing diabetic retinopathy [50]. It is therefore not necessarily specific to ITx. Furthermore, this worsening was observed in a limited number of cases (<10%), and it is difficult to determine whether islet transplantation exacerbated retinopathy or simply failed to slow its natural progression, leading to the occurrence of complications. Lastly, the precise role of immunosuppressive therapy in the progression of diabetic retinopathy remains unclear even is, based on current evidence, does not appear to exert a direct detrimental effect on the course of retinopathy.

Partial preservation of islet graft function, as opposed to complete loss of function, most likely plays a beneficial role in the progression of various diabetes-related complications. However, subgroup analyses based on the degree of graft function preservation were not available in the included studies and should therefore be further investigated in future research.

Current evidence on the impact of ITx on macrovascular diseases remain limited. Interestingly, one study suggests a higher risk of myocardial infarction in the IAK population compared to matched T1D controls. This result contrasts with other studies, which have shown improvements in cardiovascular (CV) function and reductions in CIMT. The observed increase in myocardial infarction risk should be interpreted with caution, as it may stem from limitations in the propensity score matching process. In particular, the IAK group was disadvantaged in relation to two critical CV risk factors (age and history of myocardial infarction) which may have introduced bias into the analysis [27]. Moreover, the small sample size and limited number of CV events further constrain the robustness of this association, raising the possibility of a statistically significant yet clinical uncertain finding. Two studies reported that ITx stabilizes carotid artery plaque, a key marker of cardiovascular disease [16, 22]. Improvements in left ventricular function and normalization of coagulation markers after transplantation further support its cardioprotective benefits [15].

The restoration of euglycemia after ITx is likely to play a central role, much as it does in other diabetes-related complications, in mitigating key pathophysiological processes implicated in CVD. These include chronic low-grade inflammation, oxidative stress, and endothelial dysfunction. Findings from the DCCT/EDIC study underscore this association, showing that the individuals in the intensive therapy arm experienced over a 50% reduction in strokes, non-fatal myocardial infarctions, and CV mortality, in addition to a slower CIMT progression, when compared with those receiving conventional treatment [51, 52].

Nonethless, this remains a pressing need for the future studies to explore CV outcomes in greater depth: especially concerning diabetes-related complications that have not yet been evaluated after transplantation, such as peripheral arterial disease or coronary artery disease. The application of non-invasive imaging modalities to monitor atherosclerosis progression in these contexts would be highly beneficial. Furthermore, prospective trials comparing post-transplant CV event rate (e.g., myocardial infarction, stroke) with those observed in appropriately matched T1DM controls, such as patients on the transplant waiting list, could provide more definite insights into the cardioprotective potential of ITx.

In this study, we opted for a systematic review instead of a meta-analysis due to clinical, methodological, and statistical heterogeneity. Despite this limitation, our findings clearly demonstrate the benefits of ITx in stabilizing or even reducing diabetes-related complications in individuals with T1DM.

This work has several limitations. The included studies have considerable heterogeneity, particularly regarding the prevalence and severity of each diabetes-related complication prior to transplantation. Variability is also evident in how each complication if defined and assessed, which may limit the comparability of outcomes across studies. Additionally, differences in study design further complicate interpretation. Control groups range from patients receiving intensive medical treatment to those who have undergone KTA, with considerable variation in baseline characteristics between comparator cohorts. Such methodological diversity inevitably introduces bias and complicates pooled analysis. Another relevant source of heterogeneity lies in the temporal distribution of the studies. The transplantation periods covered differ significantly, as do the immunosuppressive protocols employed. Given that immunosuppressive agents can influence the progression of certain complications, particularly nephropathy and diabetic neuropathy, this variation is of particular concern. Baseline treatment data, including nephroprotective therapies such as renin-angiotensin-aldosterone system (RAAS) inhibitors or sodium-glucose co-transporter 2 (SGLT2) inhibitors, as well as CV treatments were reported in only a very limited number of studies. Although these data are highly relevant for evaluating micro- and macrovascular complications, they were largely missing. It would therefore be beneficial for future studies reporting on the evolution of complications after ITx to include this information. Of note, the definition of each diabetes-related complication was not precisely specified in the included studies. In most cases, the focus was on the evolution of specific parameters related to the complication before and after treatment, without the use of clearly defined thresholds or cut-off values. Finally, while the available data provide a relatively robust picture of the impact of ITx on microvascular complications, the evidence concerning macrovascular outcomes remains sparse. Future research studies should prioritise targeted investigation on atherosclerosis-related conditions in transplanted patients using standardised endpoints and longitudinal follow-up to clarify the CV benefits of ITx.

This systematic review highlights a consistent improvement in microvascular complications (and possibly in macrovascular outcomes) following ITx, whether ITA or IAK (Table 2), in patients with T1DM, particularly in relation to the prevention and management of microvascular complications, and with promising indications for macrovascular outcomes. By promoting stabilisation and, in some cases, partial reversal or attenuation of disease progression, ITx emerges as a valuable therapeutic option. These findings lend further support but already strengthen the broader application of ITA or IAK for T1DM treatment not only for patients already burdened by diabetes-related complications, but also as a proactive strategy to reduce long-term morbidity and enhance quality of life in this population.

**TABLE 2:** Main findings of diabetes-related complication evolution after islets transplantation.

Author, year	Proportion of complications before tx	Baseline rate of complications	Main findings
Alhaidar [12]	Diabetic neuropathy was assessed with Utah Neuropathy Scale (UNS) and Nerve conduction study (NCV)	38.5% based on the UNS	There was no significant difference between UNS and nerve conduction study parameters at baseline and at the end of follow-up. However, a significant decrease was observed in the F-wave latencies of the peroneal nerve (50.34 ± 6.12 ms vs. 52.42 ± 6.47 ms, P = 0.005) and the ulnar nerve (27.5 ± 2.15 ms vs. 29.45 ± 2.10 ms, P = 0.009), along with an increase in ulnar sensory nerve conduction velocity (49.98 ± 6.27 m/s vs. 47.19 ± 5.36 m/s, P = 0.04)
Andres [13]	Kidney function assessed using creatinine clearance using the Cockroft-Gault formula	Basal creatinin clearance at 72 mL/min 10% with microalbuminuria and 20% with macroalbminuria	Significant decrease of ClCr from 72 mL/min to 57 mL/min
Barton [14]	Diabetic nephropathy assessed with eGFR estimated by the CKD-EPI	NA	No significant decline of eGFR
D’Addio [15]	Prothrombotic factors were assessed using thromboplastin time, prothrombin time, protein C, and protein S activated partial fibrinogen (Fg), antithrombin, fasting homocysteine d-dimer fragments (D-dimer), levels of prothrombin fragments 1 + 2 and platelet intracellular calcium using fresh plasma samples Platelet function and ultrastructure were assessed using platelet size, morphology, and granule content, and platelet areas were measured	NA	Near-normalization of platelet morphology, size, calcium platelet homeostasis and aggregation Platelets size after ITA vs. DT1 (3.199 ± 0.287 × 10^6^ nm [2] vs. 3.860 ± 0.288 × 10^6^ nm [2], p < 0.05) Levels of resting [Ca2+]I after ITA vs. DT1 (87.6 ± 18.8 nmol vs. 107.7 ± 40.0, p < 0.05) Haemostatic abnormalities with a prothrombotic state before ITA was near-normalized after islets transplantation Levels of Fg after ITA vs. DT1 (367.0 ± 26.0 mg/dL vs. 328.5 ± 35.0, p < 0.05) Levels of D-dimer after ITA vs. DT1 (0.24 ± 0.02 μg/mL vs. 1.07 ± 0.80 μg/mL)
Danielson [16]	CIMT was assessed using high-resolution B-mode carotid arteries ultrasound	Absence of cardiovascular disease	Significant reduction of CIMT at 12 months (−0.058 mm, p < 0.05) and at 50 months continued reduction of CIMT but of smaller magnitude (−0.026 mm)
Del Carro [17]	Diabetic neuropathy was assessed using NCV score, CMAP and SAP	100% of diabetic neuropathy at baseline	NCV score improved significantly in the IAK group and did not change significantly in the kidney transplant alone group Both SAP and CMAP amplitude recovered in the IAK group and worsened in the kidney transplant alone group
Deshmukh [18]	CAN was assessed using 24-h Holter monitor to evaluate Heart rate variability (HRV)	NA	No significant difference in HRV parameters at a median of 4 years between subjects post-islet transplantation and T1D individuals
Fensom [19]	NCV	66%	Subjects with diabetic neuropathy at baseline displayed significant improvement post-transplant while it worsened significantly in medically treated patients
Fiorina [20]	Kidney graft survival rate, kidney function with eGFR and urinary albumin excretion	Basal eGFR at 56.3 mL/min in the ITA group	Significant better kidney graft survival rates à 7 years in the successful IAK group 83% vs. 51%, no difference in eGFR between both group at 4 years follow-up Significant increase of microalbuminuria index in the unsuccessful IAK group (92.0 ± 64.9 to 183.8 ± 83.8, p = 0.05) and trend of reduction of microalbuminuria in the successful group (108.5 ± 53.6 to 85.0 ± 39.0)
Fiorina [21]	Hemostatic activity was assessed using prothrombin time (PT) and partial thromboplastin time (PTT), D-dimer fragments (XDP), fibrinogen (Fg), F1 2 fragments (F1 2), antithrombin III (ATIII), and protein C and S activity Patient survival and cardiovascular death were assessed	NA	In the islet transplantation group, markers of atherothrombotic risk factors were reduced. Notably, there was a significant decrease in F1.2 and XDP levels. Additionally, an improvement in natural anticoagulant activity was observed, as evidenced by increased protein C antigen activity and ATIII levels Patient survival was significantly higher in the islet transplantation group than T1D patients still on hemodialysis group (p = 0.05) The cardiovascular mortality rate was comparable with 18% in the islet transplantations, and 16% in the T1D patients still on hemodialysis group
Fiorina [22]	CIMT was assessed with high-resolution carotid arteries ultrasound Left ventricular function was measured with radionuclide left ventriculagraphy	NA	In patients who received a kidney-islet transplant, IMT remained unchanged over the 3-year follow-up period. In contrast, those in the kidney-only group experienced a significant increase in mean IMT after 3 years (p < 0.05) Significant increase in mean ejection fraction, as a marker of left ventricular systolic function, from baseline in IAK group (p < 0.05), whereas it remained unchanged in the KTA group Significant improvement in the mean peak filling rate, as a marker of left ventricular diastolic function in the IAK group (p < 0.05), while it remained unchanged in the KTA group Significant reduction of BNP from baseline in the IAK groupe and stability of BNP level in the KTA group
Hering [35]	Renal function was assessed using the Chronic Kidney Disease Epidemiology Collaboration (CKD-EPI) formula	The median eGFR at baseline was 102 mL/min/1.73m^2^	20% decline of eGFR at 2 years 102 mL/min/1.73m^2^ vs. 82 mL/min/1.73m^2^
Lee [23]	Diabetic retinopathy assessed by slit-lamp eye exam Diabetic neuropathy was assessed by electromyelogram	62.5% with diabetic retinopathy 62.5% with diabetic neuropathy	No progression of retinopathy, with one patient improving from very mild retinopathy to no retinopathy 25% showed significant improvement in conduction studies, while 75% had no notable changes in diabetic neuropathy
Lehmann [24]	Renal function was assessed by measuring serum creatinine and estimating the glomerular filtration rate (GFR) using the Chronic Kidney Disease Epidemiology Collaboration (CKD-EPI) formula	100%	There was no significant difference in the rate of decline in renal function between islet transplant recipients and pancreas transplant recipients −13.3 mL/min decline of eGFR during a follow-up of 13 years in the SIK group and 8.1% of the patients change in CKD stage during the 13 years follow-up in the SIK group
Maanoui [25]	Composite outcome, is defined as the occurrence of death, re-transplantation, or return to dialysis	Mean serum creatinine of 1.3 mg/dL eGFR (MDRD) 64.6	13 (33%) of 40 patients in the IAK transplantation group returned to dialysis or died vs. 36 (45%) in the kidney transplantation alone group
Nijhoff [34]	eGFR	NA	There was no significant change in estimated creatinine clearance, with an eGFR of 43.5 mL/min before ITx and 48.2 mL/min after ITx
O’Connell [26]	eGFR and albuminuria	0%	A 13% reduction in eGFR at 1 year (77 mL/min at baseline compared to 67 mL/min at 12 months, p = 0.051 One patient experienced an increase in their microalbuminuria level, while another developed microalbuminuria after switching to tacrolimus
Palmer [36]	CAN was assessed using resting heart rate measurements obtained from pre and post-transplant myocardial perfusionscans	NA	Significant reduction in RHR suggesting a reverse cardiovascular autonomic neuropathy after ITA
Perrier [27]	Composite criterion including, dialysis, amputation, nonfatal stroke, nonfatal myocardial infarction, transient ischemic attack and death	1.64% with amputation, 4.92% with stroke and 9.84% with myocardial infarction in the ITA group 55.6% with dialysis, 11.1% with amputation, 6.67% with stroke, 4.44% Transient ischemic attack and 13.3% with myocardial infarction in the IAK group	Compared to T1D control patients, ITA and IAK recipients had a lower risk of the composite outcome (P = 0.002 and P = 0.014, respectively), which seemed to result from lower mortality in ITA recipients (P < 0.001) and a decreased need for dialysis in IAK recipients (P < 0.001)
Rickels [28]	Kidney function was assessed with the estimated glomerular filtration rate (eGFR) derived from serum creatinine with the (CKD-EPI equation	eGFR before transplantation 100.28 mL/min/1.73m^2^ in the ITA group and 75.80 in the IAK group mL/min/	eGFR from 100.28 to 89.23 after 4.55 years of follow-up in the ITA group eGFR from 75.8 to 72.06 after 3.43 years of follow-up in the ITA group Mean UCAR was 5.6 μgm/mgm in the ITA at baseline and was 9.8 μgm/mgm after the follow-up There was a highly significant (p < 0.0001) but clinically marginal increase in UACR Mean UCAR was 26 μgm/mgm in the IAK at baseline and was 13 μgm/mgm after the follow-up There was no significant change in UCAR before in after transplantation
Ryan [29]	Renal function assessed with albuminuria Diabetic retinopathy assessed with fundoscopic examination by ophtalmologist Diabetic neuropathy was assessed with a neurothesiometer	35% with microalbuminuria 74% with diabetic retinopathy 32% with peripheral neuropathy	4 patients required retinal laser photocoagulation or vitrectomy 5 patients with microalbuminuria developed macroproteinuria Absence of progression of peripheral neuropathy
Tekin [30]	Diabetic nephropathy was assessed with eGFR and albuminuria DR was assessed with fundoscopic examination by ophtalmologist DN was assessed with NCV and clinical evaluation	None with diabetic nephropathy 50% with diabetic retinopathy 50% with diabetic neuropathy	Kidney function remained stable except in on patient who developed transient and reversible microalbuminuria No progression of diabetic retinopathy One subject developed mild axonal neuropathy within 2 years, which remained stable. Another showed no neuropathy, while two others partially improved in scores and nerve conduction
Thompson [31]	Renal assessement: eGFR assessed using MDR formula and ^99m^Tc-DTPA Retinopathy assessment: fundoscopic examination by ophtalmologist Neuropathy assessment: NCV	NA	The rate of decline of renal function is reduced in the ITA compared to the intensiveylly medical treated group Significant progression of diabetic retinopathy in the medical treated group compared to the ITA A non-significant trend toward enhanced nerve conduction velocity was observed in the ITA group
Vantyghem [32]	Diabetic neuropathy was assessed using NCV and CAN was assessed using electrophysiological and cardiovascular autonomic	NA	Significant improvement in sensory neuropathy, with no substantial changes in motor or cardiac autonomic neuropathy
Venturini [33]	DR was assessed using color Doppler imaging of the central retinal arteries and veins	80% with RD	Significant increase of blood velocities of central retinal arteries and veins in ITA patients compared to baseline before and transplantation No statistical difference at in T1D patients compared to the same group a year before Blood flow velocities of central retinal artery (psv: 6.09 ± 0.46 vs. 10.12 ± 1.20 cm/s, P < 0.01/edv: 1.65 ± 0.07 vs. 2.99 ± 0.48 cm/s, P < 0.05) Blood flow velocities of central retinal vein (maxv: 3.12 ± 0.28 vs. 6.12 ± 1.00 cm/s, P < 0.01/minv: 1.86 ± 0.22 vs. 4.14 ± 0.56 cm/s, P < 0.05)

BNP, brain natriuretic peptide; CIMT, carotid intima-media thickness test; CKD-EPI, chronic kidney disease epidemiology collaboration; CMAP, compound muscle action potential; EDV, end diastolic velocity; ESRD, end-stage renal disease; MAXV, maximum velocity; MINV, minimum velocity; NCV, nerve conduction velocyty; SAP, sensory action potential; PSV, peak systolic velocity; UTS, utah neuropathy scal.

## Data Availability

The original contributions presented in the study are included in the article/supplementary material, further inquiries can be directed to the corresponding author.

## References

[1] Nathan DM Group DER . The Diabetes Control and Complications trial/epidemiology of Diabetes Interventions and Complications Study at 30 Years: Overview. Diabetes Care (2014) 37(1):9–16. 10.2337/dc13-2112 24356592 PMC3867999

[2] Bornstein SR Ludwig B Steenblock C . Progress in Islet Transplantation Is More Important than Ever. Nat Rev Endocrinol (2022) 18(7):389–90. 10.1038/s41574-022-00689-0 35578026 PMC9109192

[3] Wisel SA Posselt AM Szot GL Nunez M Santos-Parker K Gardner JM A Multi-Modal Approach to Islet and Pancreas Transplantation with Calcineurin-Sparing Immunosuppression Maintains Long-Term Insulin Independence in Patients with Type I Diabetes. Transpl Int (2023) 36:11367. 10.3389/ti.2023.11367 37359825 PMC10285771

[4] Caldara R Tomajer V Piemonti L . Enhancing Beta Cell Replacement Therapies: Exploring Calcineurin Inhibitor-Sparing Immunosuppressive Regimens. Transpl Int (2023) 36:11565. 10.3389/ti.2023.11565 37359824 PMC10286829

[5] Berishvili E Peloso A Tomei AA Pepper AR . The Future of Beta Cells Replacement in the Era of Regenerative Medicine and Organ Bioengineering. Transpl Int (2024) 37:12885. 10.3389/ti.2024.12885 38544564 PMC10966588

[6] Gariani K Peloso A Galani V Haidar F Wassmer CH Kumar R Effect of Islet Alone or Islets After Kidney Transplantation on Quality of Life in Type 1 Diabetes: A Systematic Review. Transpl Rev (Orlando) (2024) 38(4):100870. 10.1016/j.trre.2024.100870 38917621

[7] Chetboun M Masset C Maanaoui M Defrance F Gmyr V Raverdy V Primary Graft Function and 5 Year Insulin Independence After Pancreas and Islet Transplantation for Type 1 Diabetes: A Retrospective Parallel Cohort Study. Transpl Int (2023) 36:11950. 10.3389/ti.2023.11950 38213551 PMC10783428

[8] Marfil-Garza BA Imes S Verhoeff K Hefler J Lam A Dajani K Pancreatic Islet Transplantation in Type 1 Diabetes: 20-Year Experience From a Single-Centre Cohort in Canada. Lancet Diabetes Endocrinol (2022) 10(7):519–32. 10.1016/S2213-8587(22)00114-0 35588757

[9] Harding JL Pavkov ME Magliano DJ Shaw JE Gregg EW . Global Trends in Diabetes Complications: A Review of Current Evidence. Diabetologia (2019) 62(1):3–16. 10.1007/s00125-018-4711-2 30171279

[10] Brooke BS Schwartz TA Pawlik TM . MOOSE Reporting Guidelines for Meta-Analyses of Observational Studies. JAMA Surg (2021) 156(8):787–8. 10.1001/jamasurg.2021.0522 33825847

[11] Lo CK Mertz D Loeb M . Newcastle-Ottawa Scale: Comparing Reviewers' to Authors' Assessments. BMC Med Res Methodol (2014) 14:45. 10.1186/1471-2288-14-45 24690082 PMC4021422

[12] Alhaidar M Soliven B Liao C Rubeiz H Ogledzinski M Witkowski P Long-Term Effects of Pancreatic Islet Transplantation on Polyneuropathy in Patients with Brittle Diabetes: A Single-Center Experience. Muscle Nerve (2023) 68(3):329–33. 10.1002/mus.27930 37439375 PMC10565729

[13] Andres A Toso C Morel P Demuylder-Mischler S Bosco D Baertschiger R Impact of a Sirolimus/Tacrolimus-Based Immunosuppressive Regimen on Kidney Function After Islet Transplantation. Transpl Proc (2005) 37(2):1326–7. 10.1016/j.transproceed.2004.12.040 15848711

[14] Barton FB Rickels MR Alejandro R Hering BJ Wease S Naziruddin B Improvement in Outcomes of Clinical Islet Transplantation: 1999-2010. Diabetes Care (2012) 35(7):1436–45. 10.2337/dc12-0063 22723582 PMC3379615

[15] D'Addio F Maffi P Vezzulli P Vergani A Mello A Bassi R Islet Transplantation Stabilizes Hemostatic Abnormalities and Cerebral Metabolism in Individuals With Type 1 Diabetes. Diabetes Care (2014) 37(1):267–76. 10.2337/dc13-1663 24026546 PMC3867995

[16] Danielson KK Hatipoglu B Kinzer K Kaplan B Martellotto J Qi M Reduction in Carotid Intima-Media Thickness After Pancreatic Islet Transplantation in Patients With Type 1 Diabetes. Diabetes Care (2013) 36(2):450–6. 10.2337/dc12-0679 23172970 PMC3554308

[17] Del Carro U Fiorina P Amadio S De Toni Franceschini L Petrelli A Menini S Evaluation of Polyneuropathy Markers in Type 1 Diabetic Kidney Transplant Patients and Effects of Islet Transplantation: Neurophysiological and Skin Biopsy Longitudinal Analysis. Diabetes Care (2007) 30(12):3063–9. 10.2337/dc07-0206 17804685

[18] Deshmukh T Emerson P Anderson P Kizana E O'Connell PJ Holmes-Walker DJ Cardiac Autonomic Neuropathy Is Not Reversed by Euglycemia Following Islet Transplantation. Transplantation (2021) 105(5):1125–9. 10.1097/TP.0000000000003377 32590611

[19] Fensom B Harris C Thompson SE Al Mehthel M Thompson DM . Islet Cell Transplantation Improves Nerve Conduction Velocity in Type 1 Diabetes Compared with Intensive Medical Therapy over Six Years. Diabetes Res Clin Pract (2016) 122:101–5. 10.1016/j.diabres.2016.10.011 27825059

[20] Fiorina P Folli F Zerbini G Maffi P Gremizzi C Di Carlo V Islet Transplantation Is Associated with Improvement of Renal Function Among Uremic Patients With Type I Diabetes Mellitus and Kidney Transplants. J Am Soc Nephrol (2003) 14(8):2150–8. 10.1097/01.asn.0000077339.20759.a3 12874470

[21] Fiorina P Folli F Bertuzzi F Maffi P Finzi G Venturini M Long-Term Beneficial Effect of Islet Transplantation on Diabetic Macro-/Microangiopathy in Type 1 Diabetic kidney-transplanted Patients. Diabetes Care (2003) 26(4):1129–36. 10.2337/diacare.26.4.1129 12663585

[22] Fiorina P Gremizzi C Maffi P Caldara R Tavano D Monti L Islet Transplantation Is Associated With an Improvement of Cardiovascular Function in Type 1 Diabetic Kidney Transplant Patients. Diabetes Care (2005) 28(6):1358–65. 10.2337/diacare.28.6.1358 15920052

[23] Lee TC Barshes NR O'Mahony CA Nguyen L Brunicardi FC Ricordi C The Effect of Pancreatic Islet Transplantation on Progression of Diabetic Retinopathy and Neuropathy. Transpl Proc (2005) 37(5):2263–5. 10.1016/j.transproceed.2005.03.011 15964394

[24] Lehmann R Graziano J Brockmann J Pfammatter T Kron P de Rougemont O Glycemic Control in Simultaneous Islet-Kidney Versus Pancreas-Kidney Transplantation in Type 1 Diabetes: A Prospective 13-Year Follow-up. Diabetes Care (2015) 38(5):752–9. 10.2337/dc14-1686 25665814

[25] Maanaoui M Lenain R Foucher Y Buron F Blancho G Antoine C Islet-After-Kidney Transplantation Versus Kidney Alone in Kidney Transplant Recipients With Type 1 Diabetes (KAIAK): A Population-based Target Trial Emulation in France. Lancet Diabetes Endocrinol (2024) 12(10):716–24. 10.1016/S2213-8587(24)00241-9 39250921

[26] O'Connell PJ Holmes-Walker DJ Goodman D Hawthorne WJ Loudovaris T Gunton JE Multicenter Australian Trial of Islet Transplantation: Improving Accessibility and Outcomes. Am J Transpl (2013) 13(7):1850–8. 10.1111/ajt.12250 23668890

[27] Perrier Q Jambon-Barbara C Kessler L Villard O Buron F Guerci B Impact of Islet Transplantation on Diabetes Complications and Mortality in Patients Living With Type 1 Diabetes. Diabetes Care (2025) 48:1007–15. 10.2337/dc25-0059 40245107 PMC12094206

[28] Rickels MR Eggerman TL Bayman L Qidwai JC Alejandro R Bridges ND Long-Term Outcomes With Islet-Alone and Islet-After-Kidney Transplantation for Type 1 Diabetes in the Clinical Islet Transplantation Consortium: The CIT-08 Study. Diabetes Care (2022) 45(12):2967–75. 10.2337/dc21-2688 36250905 PMC9767903

[29] Ryan EA Paty BW Senior PA Bigam D Alfadhli E Kneteman NM Five-Year Follow-Up After Clinical Islet Transplantation. Diabetes (2005) 54(7):2060–9. 10.2337/diabetes.54.7.2060 15983207

[30] Tekin Z Garfinkel MR Chon WJ Schenck L Golab K Savari O Outcomes of Pancreatic Islet Allotransplantation Using the Edmonton Protocol at the University of Chicago. Transpl Direct (2016) 2(10):e105. 10.1097/TXD.0000000000000609 27795987 PMC5068201

[31] Thompson DM Meloche M Ao Z Paty B Keown P Shapiro RJ Reduced Progression of Diabetic Microvascular Complications with Islet Cell Transplantation Compared with Intensive Medical Therapy. Transplantation (2011) 91(3):373–8. 10.1097/TP.0b013e31820437f3 21258272

[32] Vantyghem MC Quintin D Caiazzo R Leroy C Raverdy V Cassim F Improvement of Electrophysiological Neuropathy After Islet Transplantation for Type 1 Diabetes: A 5-Year Prospective Study. Diabetes Care (2014) 37(6):e141–142. 10.2337/dc14-0320 24855172

[33] Venturini M Fiorina P Maffi P Losio C Vergani A Secchi A Early Increase of Retinal Arterial and Venous Blood Flow Velocities at Color Doppler Imaging in Brittle Type 1 Diabetes After Islet Transplant Alone. Transplantation (2006) 81(9):1274–7. 10.1097/01.tp.0000208631.63235.6a 16699454

[34] Nijhoff MF Engelse MA Dubbeld J Braat AE Ringers J Roelen DL Glycemic Stability Through Islet-After-Kidney Transplantation Using an Alemtuzumab-Based Induction Regimen and Long-Term Triple-Maintenance Immunosuppression. Am J Transpl (2016) 16(1):246–53. 10.1111/ajt.13425 26288226

[35] Hering BJ Clarke WR Bridges ND Eggerman TL Alejandro R Bellin MD Phase 3 Trial of Transplantation of Human Islets in Type 1 Diabetes Complicated by Severe Hypoglycemia. Diabetes Care (2016) 39(7):1230–40. 10.2337/dc15-1988 27208344 PMC5317236

[36] Palmer JBA MacIsaac A Goodman D . Islet Cell Transplantation Lowers Resting Heart Rate in Type 1 Diabetics Suggesting Improved Cardiovascular Autonomic Neuropathy. Heart Lung Circ (2021) 30:S159. 10.1016/j.hlc.2021.06.150

[37] Fiorina P Folli F Maffi P Placidi C Venturini M Finzi G Islet Transplantation Improves Vascular Diabetic Complications in Patients With Diabetes Who Underwent Kidney Transplantation: A Comparison Between Kidney-Pancreas and Kidney-Alone Transplantation. Transplantation (2003) 75(8):1296–301. 10.1097/01.TP.0000061788.32639.D9 12717219

[38] Johansson BL Borg K Fernqvist-Forbes E Kernell A Odergren T Wahren J . Beneficial Effects of C-Peptide on Incipient Nephropathy and Neuropathy in Patients With Type 1 Diabetes Mellitus. Diabet Med (2000) 17(3):181–9. 10.1046/j.1464-5491.2000.00274.x 10784221

[39] Brunskill NJ . C-peptide and Diabetic Kidney Disease. J Intern Med (2017) 281(1):41–51. 10.1111/joim.12548 27640884

[40] Sjoquist M Huang W Johansson BL . Effects of C-peptide on Renal Function at the Early Stage of Experimental Diabetes. Kidney Int (1998) 54(3):758–64. 10.1046/j.1523-1755.1998.00074.x 9734600

[41] Johansson BL Kernell A Sjoberg S Wahren J . Influence of Combined C-peptide and Insulin Administration on Renal Function and Metabolic Control in Diabetes Type 1. J Clin Endocrinol Metab (1993) 77(4):976–81. 10.1210/jcem.77.4.8408474 8408474

[42] The Effect of Intensive Diabetes Therapy on the Development and Progression of Neuropathy. The Diabetes Control and Complications Trial Research Group. Ann Intern Med (1995) 122(8):561–8. 10.7326/0003-4819-122-8-199504150-00001 7887548

[43] Boulton AJ Malik RA Arezzo JC Sosenko JM . Diabetic Somatic Neuropathies. Diabetes Care (2004) 27(6):1458–86. 10.2337/diacare.27.6.1458 15161806

[44] Ekberg K Brismar T Johansson BL Jonsson B Lindstrom P Wahren J . Amelioration of Sensory Nerve Dysfunction by C-Peptide in Patients with Type 1 Diabetes. Diabetes (2003) 52(2):536–41. 10.2337/diabetes.52.2.536 12540632

[45] Sima AA Zhang W Sugimoto K Henry D Li Z Wahren J C-Peptide Prevents and Improves Chronic Type I Diabetic Polyneuropathy in the BB/Wor Rat. Diabetologia (2001) 44(7):889–97. 10.1007/s001250100570 11508275

[46] Cotter MA Ekberg K Wahren J Cameron NE . Effects of Proinsulin C-peptide in Experimental Diabetic Neuropathy: Vascular Actions and Modulation by Nitric Oxide Synthase Inhibition. Diabetes (2003) 52(7):1812–7. 10.2337/diabetes.52.7.1812 12829651

[47] Arnold R Issar T Krishnan AV Pussell BA . Neurological Complications in Chronic Kidney Disease. JRSM Cardiovasc Dis (2016) 5:2048004016677687. 10.1177/2048004016677687 27867500 PMC5102165

[48] Sorrentino FS Matteini S Bonifazzi C Sebastiani A Parmeggiani F . Diabetic Retinopathy and Endothelin System: Microangiopathy Versus Endothelial Dysfunction. Eye (Lond). (2018) 32(7):1157–63. 10.1038/s41433-018-0032-4 29520046 PMC6043602

[49] The Absence of a Glycemic Threshold for the Development of Long-Term Complications: The Perspective of the Diabetes Control and Complications Trial. Diabetes (1996) 45(10):1289–98. 10.2337/diab.45.10.1289 8826962

[50] Bain SC Klufas MA Ho A Matthews DR . Worsening of Diabetic Retinopathy with Rapid Improvement in Systemic Glucose Control: A Review. Diabetes Obes Metab (2019) 21(3):454–66. 10.1111/dom.13538 30226298 PMC6587545

[51] Nathan DM Cleary PA Backlund JY Genuth SM Lachin JM Orchard TJ Intensive Diabetes Treatment and Cardiovascular Disease in Patients With Type 1 Diabetes. N Engl J Med (2005) 353(25):2643–53. 10.1056/NEJMoa052187 16371630 PMC2637991

[52] Nathan DM Lachin J Cleary P Orchard T Brillon DJ Backlund JY Intensive Diabetes Therapy and Carotid Intima-Media Thickness in Type 1 Diabetes Mellitus. N Engl J Med (2003) 348(23):2294–303. 10.1056/NEJMoa022314 12788993 PMC2701300

